# Degradation of Residual Herbicide Atrazine in Agri-Food and Washing Water

**DOI:** 10.3390/foods11162416

**Published:** 2022-08-11

**Authors:** Junting Hong, Nadia Boussetta, Gérald Enderlin, Franck Merlier, Nabil Grimi

**Affiliations:** 1 Université de Technologie de Compiègne, ESCOM, TIMR (Integrated Transformations of Renewable Matter), Centre de Recherche Royallieu, CEDEX CS 60319, 60203 Compiègne, France; 2 Université de Technologie de Compiègne, UPJV, CNRS, Enzyme and Cell Engineering, Centre de Recherche Royallieu, CEDEX CS 60319, 60203 Compiègne, France

**Keywords:** atrazine, degradation, residue, agri-food, water

## Abstract

Atrazine, an herbicide used to control grassy and broadleaf weed, has become an essential part of agricultural crop protection tools. It is widely sprayed on corn, sorghum and sugar cane, with the attendant problems of its residues in agri-food and washing water. If ingested into humans, this residual atrazine can cause reproductive harm, developmental toxicity and carcinogenicity. It is therefore important to find clean and economical degradation processes for atrazine. In recent years, many physical, chemical and biological methods have been proposed to remove atrazine from the aquatic environment. This review introduces the research works of atrazine degradation in aqueous solutions by method classification. These methods are then compared by their advantages, disadvantages, and different degradation pathways of atrazine. Moreover, the existing toxicological experimental data for atrazine and its metabolites are summarized. Finally, the review concludes with directions for future research and major challenges to be addressed.

## 1. Introduction

**Figure 1:**
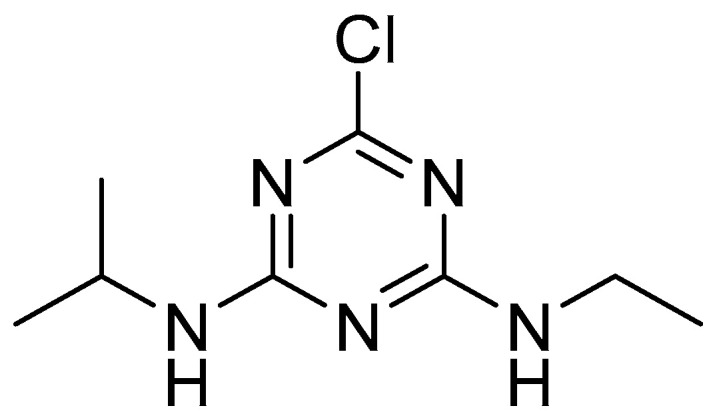
Atrazine (2-chloro-4-ethylamino-6-isopropylamino-1,3,5-triazine).

Atrazine (Figure 1) is a triazine herbicide with a wide range of applications, for grassy and broadleaf weed control in corn, sugarcane, sorghum and certain other crops [1,2,3,4]. Due to of its efficiency and low cost, its average consumption worldwide is 70,000 to 90,000 tons per year [5]. If shopping for conventional groceries, consumers are likely to have eaten food that has been sprayed with atrazine. Since atrazine is applied to crops used as livestock feed, its residues are found not only in crops, but also in milk and meat. According to the consumer risk assessment performed by the European Food Safety Authority [6], atrazine input values used for the dietary chronic exposure calculation of maize and other cereals except maize are 0.025 mg/kg and 0.05 mg/kg, respectively, based on the mean consumption data representative for 22 national diets. Although not considered acutely toxic to people, atrazine affects long term human health. Atrazine can act as the endocrine disrupting chemicals (EDC) [7] that can produce damage to the endocrine system, and cause a series of pathological changes and reproductive abnormalities [8]. Additionally, atrazine is also a potential carcinogen due to negative impact on human health such as tumors, breast, ovarian, and uterine cancers as well as leukemia and lymphoma [9]. For these reasons, atrazine was banned in the European Union (EU) in 2003 [10]. However, the commercial formulations of the herbicide atrazine (such as Gesaprim 90% WG) are still widely employed in Latin America. For example, herbicides were the main pesticide class used in Brazil between 2009 and 2018, with oscillations from 52.4% (2011) to 62.5% (2012), and atrazine was the top two active ingredient in this period [11]. Brazil is the world’s third biggest exporter of agricultural products and organic food market leader in Latin America [12]. In addition, Brazil’s main export markets are the European Union and the United States [13]. So, the residual problem of atrazine still remains a concern. Atrazine is chemically stable with long half-life in water (30–100 days) [14,15], and its microbial degradation in soil environments is a relatively slow process (the range of field half-lives is 18 to 148 days [16,17]). It is also slightly soluble in water (33 mg·L^−1^ at 22 °C) and has low adsorption in soil [18]. Thus, it contaminates both surface and ground water [19]. The upper limit for atrazine in drinking water is 3 μg/L in America whereas in Europe, it is fixed as 1 μg/L [20,21]. However, investigations [22,23,24] have shown that concentrations of atrazine exceed the authorized limit of water contamination in surface water and ground water. Lots of works [25,26,27,28] have been conducted on the detection and quantification of atrazine in water, which is important to the food safety and quality control. Controlling the pollution of residual atrazine in agri-food and washing water has become a major issue.

So far, many treatment technologies of aqueous atrazine have been developed, including microwave assisted photo reactions, advanced oxidation processes (AOPs), bioremediation, etc. This review summarizes recent degradation progress of atrazine in water, with an emphasis on current chemical methods (Fenton/Fenton-like Method [29,30,31,32,33], Sulfate Radical Oxidation [34,35,36,37,38], Photocatalytic Method [39,40,41,42,43], Electrocatalytic Method [44,45,46,47,48], Ozone Oxidation Method [49,50,51,52,53]), Biodegradation (Microbial Degradation [54,55,56,57,58] and Phytodegradation [59,60,61,62,63]) and physicochemical methods (High Voltage Electrical Discharges [64,65,66,67,68,69], Ultrasound [70,71,72], Microwave [73,74,75] and Ionizing Radiation [76,77,78]). Although two recently published reviews [79,80] also describe atrazine degradation techniques, they do not cover degradation methods comprehensively. This review not only expands the atrazine degradation techniques, but also compares them in terms of degradation pathways, atrazine mineralization, and metabolite toxicity.

## 2. Chemical Method

### 2.1. Fenton/Fenton-like Method

The classical Fenton reaction describes the activation of hydrogen peroxide ($H_{2}O_{2}$) by ferrous ($Fe^{2+}$) ions to generate hydroxyl radicals (HO) [3]. The hydroxyl radical abstracts a hydrogen atom from organic substrate (R−H), and generates an organic radical (R), which subsequently undergoes a series of chemical transformation to form various oxidation products. The reactions are as follows:

(1) $$Fe^{2+}+H_{2}O_{2}\to \;Fe^{3+}+HO\cdot +OH^{-}$$

(2) $$RH+HO\cdot \;\to \;H_{2}O+R\cdot \;\to further\;oxidation$$

Although the classic Fenton oxidation achieves the generation of free radicals and has strong oxidizing ability under ambient conditions, insoluble ferric hydroxide precipitates are generated during the process, which reduces the overall oxidation efficiency and requires continuous addition of $Fe^{2+}$ salt. Therefore, Fenton-like methods with higher oxidation efficiency have been developed. For example, photo-Fenton, electro-Fenton and sono-Fenton are improvements of Fenton oxidation combined with photochemistry, electrochemistry, and ultrasound, respectively, and they have been used for the degradation of aqueous atrazine. In 2002, Venturaet et al. [29] designed an electro-Fenton system and used it for the degradation of atrazine. The electro-Fenton system could continuously produce the ferrous iron and the hydrogen peroxide, thereby allowing more efficient generation of ·OH, which led to a more thorough oxidation of atrazine. In the same year, Saltmiras et al. [30] published a similar work using anodic Fenton treatment to degrade 70% of atrazine in 3 min.

In 2020, Yang et al. [81] prepared a heterogeneous Fenton catalyst $Fe/TiO_{2}$ using $TiO_{2}$ synthesized by sol-gel method as carrier and ferric nitrate as Fe source, which could effectively remove atrazine under visible light, achieving over 95% removal efficiency within 30 min. In 2020, Shi et al. [82] reported $Fe_{3}S_{4}$ Fenton oxidation of atrazine using visible light, and atrazine was completely degraded within 35 min. In 2021, Fareed et al. [83] adopted the UV/$FeCl_{3}$/$H_{2}O_{2}$ system and achieved a 97% degradation rate of atrazine. In addition, the use of iron-modified mesoporous molecular sieve materials to degrade atrazine using UV–vis irradiation was reported by Benzaquén et al. [84]. Additionally, there are other related photo-Fenton systems, using tantalum (oxy)nitrides to prepare photocatalytic materials, on the degradation of aqueous atrazine [32,33,85,86].

The stepwise-Fenton’s processes for the degradation of atrazine were developed by Chu et al. in 2007 [31]. And according to the system models built through the examination of reaction kinetics, they found that the performance of stepwise- Fenton’s processes was better than that of conventional Fenton’s processes.

### 2.2. Sulfate Radical ($SO_{4}^{-}$) Oxidation Method

Compared with OH, the sulfate radical $SO_{4}^{-}$ has a higher redox potential, longer half-life, and higher selectivity for electron transport reactions, receiving increasing attention on the degradation of pollutants [34]. So far, there are many generation methods of $SO_{4}^{-}$ for atrazine removal (Table 1).

In addition, there are processes that combine sulfate radical oxidation with other technologies such as UV-vis [97,98]. The photocatalysis technology is needed for the activation of sulfite to generate $SO_{4}^{-}$ effectively at the neutral pH condition without any precipitation of metal-hydroxyl species, thus greatly improving the degradation rate of atrazine.

### 2.3. Photocatalytic Method

**Figure 2:**
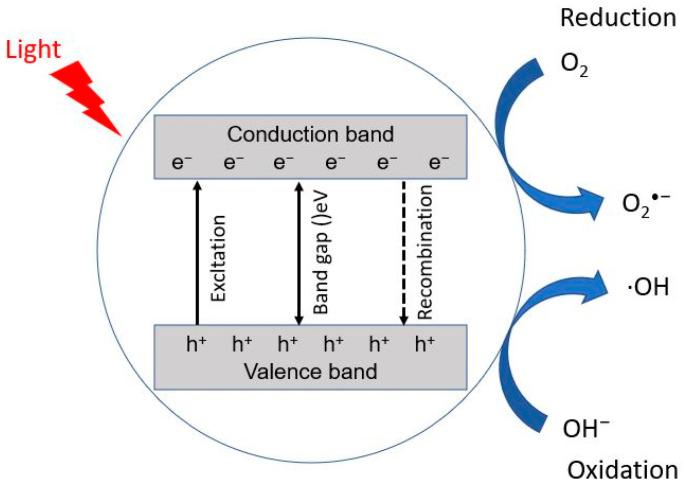
Schematic representation of mechanism of photocatalysis.

Photocatalysis generally refers to a photochemical reaction with the participation of a catalyst. Under the irradiation of ultraviolet or visible light, electron–hole pairs are created by photocatalysts, which generate free radicals such as OH able to oxidize and decompose organic pollutants. The image below (Figure 2) refers to reference [99].

The general photocatalysts are N-type semiconductor materials, which have the characteristics of low band gap, such as $TiO_{2}$,$\;ZrO_{2}$, ZnO, CdS,$\;WO_{3}$, $Fe_{2}O_{3}$, $Bi_{2}O_{3}$, etc. Among them, Ti-based, W-based, and Bi-based materials and their oxides are commonly used in the photodegradation of aqueous atrazine (Table 2).

In addition, photoelectrocatalysis (PEC), which combines both electrochemistry and photocatalysis, has also used in the degradation of aqueous atrazine. In 2018, Fernández-Domene et al. [100] reported the degradation of atrazine by photo-electrocatalysis using a photoanode based on $WO_{3}$ nanosheets. And atrazine was completely degraded after 180 min. In 2021, Xie et al. [101] used the bias potential applied on the photo-anode to achieve a 96.8% removal efficiency of atrazine.

The photocatalytic method has received widespread attention because of its high efficiency, non-toxicity, and lack of secondary pollution. It is recommended to use visible light catalytic process to degrade atrazine, because the use of solar energy is sustainable and environmentally friendly.

**Table 2:** Photodegradation of aqueous atrazine.

Photocatalyst	Preparation	Light Source	Removal Effect
In, S-TiO_2_@rGO nanocomposite	TiO_2_@rGO nanocomposites were synthesized based on a new ultrasonic-assisted hydrothermal method.	Visible-light, a 300 W tungsten xenon lamp.	The complete degradation and 95.5% mineralization of atrazine was achieved within 20 min [39].
Boron-doped TiO_2_	Used a one-step calcination method.	Visible-light, a 350 W (15 A) Xenon lamp with a 300 nm cutoff filter (CHF-XM-350 W, Beijing Trusttech. Co., Beijing, China).	The degradation of atrazine was up to 95% [40].
Metalloporphyrins supported on TiO_2_	Tetra (4-carboxyphenyl) porphyrin with different metal centers and metal-free was adsorbed on TiO_2_ surface.	Visible-light, an open borosilicate (Pyrex) glass cell with an optical window of 11 cm^2^ area.	82% of atrazine was degraded using Cu(II) porphyrin within 1 h [41].
Crystal TiO_2_ nanowires with high specific surface area	Use a PEG-assisted hydrothermal method.	UV irradiation, two 15 W Philips UV light lamps (365 nm wavelength, intensity: 2.47 ± 0.16 mW cm^−2^).	The degradation of atrazine is up to 60% in 1 h [42].
TiO_2_ nanoparticles involved boron enrichment waste		UV irradiation, a UV lamp (400 W, λ = 250–570 nm).	The degradation of atrazine is up to 60% in 70 min. The removal of atrazine followed a pseudo-first-order reaction kinetic [43].
Mesoporous Ag-WO_3_/SBA-15 composite		Visible-light, a broadband light source (450 W Xe arc lamp) fitted with a neutral density optical filter to allow light of wavelength above 400 nm.	70% of atrazine was degraded in 18 min [102].
Heterojunction BiVO_4_-Bi_2_O_3_	Platelet-like BiVO_4_ was synthesized by hyperbranched polyethyleneimine [103].	Visible-light, a mercury 250 W High-Pressure lamp.	The heterojunction efficiently removed >90% of atrazine [104].
CdS/BiOBr/Bi_2_O_2_CO_3_ ternary heterostructure materials	Used a simple one pot hydrothermal method.	Visible-light, a 250 W xenon lamp with a 400 nm cutoff filter.	The degradation of atrazine was up to 95% in 30 min [105].
BiOBr/UiO-66 composite	Used an in situ growth method.	Visible-light, a 300 W Xe lamp (Beijing Zhongjiaojinyuan, CEL-HXF300) with a 400 nm cut-off glass filter.	The degradation of atrazine was up to 90% in 3 h [106].
Cu-BiOCl	Used a one-pot solvothermal method.	UV irradiation, a Steripen Mercury UV lamp with emission wavelength of 254 nm.	29% of atrazine was degraded [107].

### 2.4. Electrocatalytic Method

Electrocatalysis is a catalytic process involving oxidation and reduction reactions through the direct transfer of electrons, which requires electrocatalysts to lower the overpotential of the reactions [108]. Electrocatalytic oxidation technology can produce ∙OH in situ and no additional chemical reagent is required, which can remove atrazine from wastewater efficiently and environmental-friendly [44]. Electrode materials play an essential role in the progress of electrocatalytic oxidation. Various types of electrodes have been exploited for the degradation of atrazine in water (Table 3).

In addition, electrochemistry has also been combined with ozone oxidation to degrade aqueous atrazine [110]. In 2016, Zhou et al. proposed a novel oxidation process using iron electrodes and ozone in atrazine degradation [111]. Moreover, atrazine degradation by in situ electrochemically generated ozone was reported by Vera et al. in 2009 [112]. The combination of electrochemistry and ozonation exhibited higher removal efficiency for ATZ than ozonation and electrocoagulation [111].

Moreover, Electrochemical Advanced Oxidation Processes (EAOPs) is also an efficient method to remove recalcitrant molecules. Atrazine is a very stable molecule with a relative resistance to microbial attack. Therefore, EAOPs can be used for pretreatment, before the biodegradation of atrazine [109].

### 2.5. Ozone Oxidation Method

Ozone is a strong oxidant, which can oxidize organic or inorganic substances in wastewater, thereby disinfecting, oxidizing or decolorizing. Because atrazine is resistant to the degradation by ozone, additional catalysts are required for the ozonation of atrazine [113]. In recent years, the ozonation of aqueous atrazine has been reported (Table 4).

In addition, using ozone oxidation combined with other oxidation processes can improve the degradation efficiency and mineralization rate of atrazine. In 2006, Bianchi et al. [114] studied the mechanism of atrazine degradation in aqueous phase under sonolysis at 20 kHz, ozonation, photolysis at 254 nm and photocatalysis in the presence of TiO_2_, employed either separately or in combination. Ozonation and photocatalysis induced atrazine de-alkylation, followed by slower de-chlorination, and simultaneous sonolysis increased the rate of photocatalytic de-alkylation. The highest degradation rate of atrazine was achieved when photolysis at 254 nm was combined with ozonation.

**Table 4:** Ozonation of aqueous atrazine.

Catalyst	Removal Effect
Manganese	The presence of humic substances has a substantial influence on the Mn-catalysed ozonation of atrazine [49].
A non-ionic surfactant, Brij35 (polyoxyethylene (23) lauryl ether)	Atrazine was completely removed after a reaction time of 2 h [50].
Nano-ZnO	The degradation efficiency of atrazine was 99% after 5 min reaction at pH 6 [51].
Mesoporous Fe_3_O_4_	The removal rate of atrazine was up to 97% [52]
Hydroxylamine	80% of atrazine was degraded by ozonation in the presence of hydroxylamine [53].
Rutile TiO_2_	The removal rate and the mineralization of atrazine was 93% and 56%, respectively [115].
Oxygen functionalized graphitic carbon nitrideO@g-C_3_N_4_	The removal rate of atrazine was 93%, after 5 min reaction at pH 6 [116].
Three-dimensional Co/Ni bimetallic organic frameworks	94% of atrazine were removed [117].

## 3. Biodegradation

Biodegradation refers to the partial, and sometimes total, transformation or detoxification of contaminants by microbial, plants or enzymes [118]. It has advantages over physical and chemical methods in terms of low costs and environmental friendliness [119]. Since the discovery of biotic atrazine degradation [120,121], biodegradation has been a major method for atrazine catabolism [1].

### 3.1. Microbial Degradation

Microbial degradation exploits the ability of microorganisms for removal of pollutants from contaminated sites [122]. That is because indigenous microorganisms that are already present in polluted environments may transform pollutants to harmless products via reactions that take place as a part of their metabolic processes [123]. Generally, isolated microbes are selected for the degradation due to nature and type of pollutants. Different atrazine-degrading bacteria and fungi have been isolated (Table 5). Because microorganisms are easily drained in water making their effectiveness greatly reduced, Yu et al. [58] developed a self-immobilized biomixture (SIB) with biosorption and biodegradation properties, that can obtain better atrazine removal rate.

### 3.2. Phytodegradation

The phytodegradation of organic compounds take place inside the plant or within the rhizosphere of the plant [126]. Rhizosphere, the immediate vicinity of plant roots, is a zone of intense microbial activity, and the use of vegetation at the waste sites can overcome the inherent limitations such as low microbial population or inadequate microbial activity [59]. It has been reported that atrazine can be degraded or detoxified in crops [60,61], and the molecular mechanism for catabolism and detoxification of atrazine in plants is a major research topic (Table 6).

Generally, atrazine may be degraded within the plant biomass by plant enzymes as well as in its rhizosphere by microbial biotransformation [127,128].

## 4. Physicochemical Method

### 4.1. High Voltage Electrical Discharges (HVED)

High Voltage Electrical Discharges (HVED) is one of the advanced oxidation processes that has been used for the treatment of wastewater. During the discharge processes of gas and liquid system, the low-temperature plasma, high-energy electrons and UV-radiation are generated to degrade wastewater. The generated plasma is a conductive fluid that is electrically neutral and consists of electrons, positive and negative ions, free radicals, neutral particles and excited-state atoms [129]. Among them, the high-energy electrons bombard water molecules to ionize and generate oxidants such as ·OH and $H_{2}O_{2}$, which can efficiently degrade organic substances. The main reactions include:

(3) $$H_{2}O\to H\cdot +\cdot OH$$

(4) $$H\cdot +O_{2}\;\to HO_{2}\cdot$$

(5) $$H\cdot +HO_{2}\cdot \;\to \;H_{2}O_{2}$$

(6) $$H_{2}O_{2}+e\;\to \;\cdot OH+OH^{-}$$

(7) $$RH+\cdot OH\to R\cdot +H_{2}O$$

The plasma reactors can be divided into three types. One is the non-thermalizing electrical discharge applied in the air above an aqueous solution, generating an atmospheric plasma. The second is the discharge applied into the water, creating high-temperature plasma channels. In addition, the hybrid reactors utilize both gas phase nonthermal plasma formed above the water solution and direct liquid phase corona-like discharge in water [130].

In 1997, Houben et al. [64] reported a research work on the degradation of atrazine by pulsed corona discharges above the water surface, in which 0.12 mM atrazine was oxidized for 5 h and the degradation rate was 57%. This is the earliest work using plasma reactors to degrade atrazine. Several years later, in 2005, Karpel Vel Leitner et al. [65] applied the pulsed arc electrohydraulic discharge (PAED) system on the degradation of atrazine. PAED was generated by a spark gap type power supply (0.5 kJ/pulse) with rod-to-rod type electrodes in water. The removal rate of atrazine (0.5 μmol/L) achieved 80% with inter-electrode gap of 4 mm when the input energies were higher than 10 kJ/L. In 2007, Mededovic and Locke [66] present an investigation of the atrazine degradation by pulsed electrical discharge in water. Different electrolytes and electrode materials were studied. An initial pH 3 (adjusted with H_2_SO_4_) 90% of the atrazine (2 × 10^−5^ M) was degraded in 1 h, and the final degradation product was ammeline. When ferrous ions were used as an electrolyte, atrazine was degraded within 10 min due to the hydrogen peroxide produced by the discharge which reacted with ferrous ions. In addition, they compared their work with the above two pulsed electrical discharge works. The comparison of energy efficiency showed that the underwater pulsed electrical discharge had higher atrazine conversion for the same energy input than discharge above the water surface and pulsed arc discharge (Table 7).

Moreover, there are four other works using dielectric barrier discharge (DBD), a typical non-equilibrium high-voltage gas discharge. In 2014, Zhu et al. [67] designed a novel wire–cylinder DBD plasma reactor for atrazine degradation, and the degradation rate was up to 93.7%, and 12.7% of total organic carbon (TOC) was removed after 18 min of discharge at the optimum conditions (input power = 50 W, air flow rate = 140 L·h^−1^). In 2015, Patrick Vanraes et al. combined DBD with absorption of activated carbon [69] or nanofiber membrane [68] on the degradation of atrazine. In 2021, Wang et al. [131] combined DBD with microbubbles (MBs) for persulfate (PS) activation and atrazine removal in water. Under these DBD/MBs/PS systems, the degradation efficiency reached 89% after 75 min of treatment at a discharge power of 85 W, a PS concentration of 1 mM, and an air flow rate of 30 mL/min. And according to the calculated energy yield (EY 41.8 mg/kWh at a discharge power of 85 W), they supposed that DBD/MBs/PS system was economically viable in treating large scale atrazine wastewater.

In addition, there is another report on the remediation of atrazine in a plasma reactor. In 2018, Aggelopouloset al. [132] used DBD plasma at atmospheric air pressure to treat a sandy soil polluted with atrazine. The atrazine degradation rates of 87% and 98% were achieved after 60 min of plasma treatment, starting from initial pollutant concentrations of 100 and 10 mg/kg, respectively.

HVED is an innovative technique, which combines sonochemistry, high-energy electron radiation, photochemistry, etc., and can effectively decompose organic pollutants. Nevertheless, the use of HVED for wastewater treatment is still under development, and further research is needed. The research on the degradation behavior of aqueous atrazine by plasma deserves more attention.

### 4.2. Ultrasound

The main principles of ultrasonic degradation of pollutants in water are cavitation effect and free radical oxidation. The high energy generated by the collapse of the ultrasonic cavitation bubble is sufficient to break the chemical bond and generate hydroxyl radicals ·OH and hydrogen radicals ·H, which oxidize organic substances and transform into $CO_{2}$, $H_{2}O$, inorganic ions or low-toxic organic compounds. At the same time, the rupture of bubbles enhances the purification. In wastewater treatment, ultrasound technique is often combined with other techniques [133] (ozone oxidation, ultraviolet irradiation, biodegradation, etc.) to achieve efficient degradation.

The earliest report on ultrasonic treatment of aqueous atrazine was reported by W.C. Koskinen et al. in 1994 [134], and the kinetic of sonochemical decomposition of atrazine in water was determined. In 1996, Petrier et al. [70] used two frequencies (20 kHz and 500 kHz) to degrade atrazine in aqueous solution. The degradation rate of atrazine was nearly 100% after 80 min at 500 kHz and 55% after 120 min at 20 kHz.

Later, ultrasonic treatment was combined with other techniques to degrade aqueous atrazine, and it is common to combine US and UV, or US and ozonation. In 2001, A. Hiskia et al. [135] published a report on US/UV decomposition of atrazine in the presence of polyoxometalates (POM) within a few minutes, giving common intermediates, namely, 2-hydroxy-4-(isopropylamino)-6-(ethylamino)-s-triazine (HA), 2-chloro-4-(isopropylamino)-6-amino-s-triazine (DEA), 2-chloro-4-amino-6-(ethylamino)-s-triazine (DIA), ammeline (AM) among others. The final products for both methods, US and UV with POM, were cyanuric acid, NO_3_^−^, Cl^−^, CO_2_, and H_2_O. In 2012, R. Kidak and S. Dogan [136] investigated the efficiency of O_3_ and US and also of their combined application (US + O_3_) for the degradation and potential mineralization of atrazine in water, leading to 95% removal for O_3_ and 78% for US after 90 min of treatment, and 100% for US + O_3_ after 20 min of treatment. In 2014, Xu et al. [71] reported sonophotolysis (US/UV) for the degradation of atrazine. After 60 min of sonophotolysis treatment, the complete degradation of atrazine and 60% total organic carbon (TOC) removal rate were achieved. In 2017, Jing et al. [72] used a pilot-scale UV/O_3_/US flow-through system to remove atrazine from wastewater. The optimal atrazine removal rate (98%) was obtained at the conditions of 75 W UV power, 10.75 g·h^−1^ O_3_ flow rate and 142.5 W ultrasound power.

Ultrasonic treatment has a strong effect on the degradation of organic substances, but it has the problem of high energy consumption. For the degradation of aqueous atrazine, more consideration can be given to combine ultrasonic treatment with other techniques, such as biodegradation, electrochemistry, Fenton oxidation, etc.

### 4.3. Microwave

Microwave treatment is a breakthrough, innovative, and broad-spectrum water treatment technique. It achieves the effect of decontamination and sterilization through the selective heating, low-temperature catalysis, and rapid penetration by the microwave field. The principle is that microwave heating generates efficient internal heat-transfer by penetrating subjects and causing uniform energy distribution throughout the material irradiated, which leads to an even chemical reaction [137]. Microwave irradiation can cause atrazine degradation through formation of micro-scale “hot spots” on the pore wall surface that pyrolyze the absorbed organic molecules [138].

In existing reports, microwave is often used as an auxiliary technique for the treatment of atrazine. The earliest work was on the microwave-assisted extraction of atrazine from soil, reported by Xiong et al. [73] in 1998. The combination of microwave (MW) power and ultraviolet (UV) light can improve the photochemical process, thereby making the degradation of atrazine more efficient. In 2006, Ta et al. [74] reported the degradation of atrazine by microwave-assisted electrode less discharge mercury lamp (MW-EDML) in aqueous solution. Microwave improved the photolysis of atrazine under UV-vis irradiation, so that it was completely degraded in a relatively short time (i.e., *t*_1/2_ = 1.2 min for 10 mg/L). Additionally, the main degradation products during atrazine degradation process were identified by gas chromatography mass spectrometry (GC–MS) and liquid chromatography mass spectrometry (LC–MS), according to which the degradation mechanism including four possible pathways for atrazine degradation was proposed. In 2007, Gao et al. [75] reported a method of microwave-assisted photocatalysis on $TiO_{2}$ nanotubes for the degradation of aqueous atrazine. Atrazine was completely degraded in 5 min and the mineralization efficiency was 98% in 20 min, which superior to many other atrazine degradation works (they cannot achieve complete atrazine degradation with the formation of many toxic intermediates such as Deethylatrazine, Deisopropylatrazine, ammeline, etc.). High mineralization efficiency means that atrazine was released in soluble inorganic forms such as CO_2_, H_2_O, NH_4_^+^ and small acids, which is beneficial to the non-toxic treatment of wastewater. Therefore, for the degradation of atrazine, not only a high degradation efficiency, but also a high mineralization rate is very important. In 2011, Chen et al. [139] used a microwave photochemical reactor to degrade atrazine in the presence of hydrogen peroxide $H_{2}O_{2}$. The optimal condition of atrazine degradation by MW/UV/H_2_O_2_ process was 53 °C, 300 mg/L H_2_O_2_, MW power *P*_appl_ = 30 ± 0.3 W (half-life *t*_1/2_ = 1.1 min for 20.8 mg/L initial concentration). Comparing with other processes such as UV alone [139] (half-life *t*_1/2_ = 9.9 min for 20 mg/L initial concentration), UV/H_2_O_2_ [140] (half-life *t*_1/2_ = 1.2 min for 8.4 mg/L initial concentration with 343.4 mg/L H_2_O_2_) and MW/UV [139] (half-life *t*_1/2_ = 2.2 min for 20.8 mg/L initial concentration), microwave-assisted photocatalytic method is better than traditional photocatalytic methods, and adding H_2_O_2_ can achieve high-efficiency degradation of aqueous atrazine.

In addition, for traditional adsorption, its degradation efficiency highly depends on the adsorbent, while microwave heating can modify the adsorbent to bring about highly efficient adsorbent performance. Therefore, the adsorption and degradation of aqueous atrazine under microwave heating has attracted attention. Hu et al. [138,141] reported the adsorption and degradation of atrazine in transition metal-loaded microporous under microwave induction. In 2017, Wei et al. [142] enhanced adsorption of atrazine using a coal-based activated carbon modified with sodium dodecyl benzene sulfonate under microwave heating. In the same year, Sivarajasekar et al. reported a fixed-bed column towards sorptive removal of Atrazine from aqueous solutions using microwave irradiated Aegle marmelos Correa fruit shell.

### 4.4. Ionizing Radiation (γ-Rays, Electron Beams)

In recent years, due to environmental protection, ionizing radiation treatment of pollutants has received more and more attention. Ionizing radiation can cause displacement of electrons from atoms and breaks in chemical bonds, and γ-rays and electron beams are most commonly employed forms [143].

In 2009, Basfar et al. [76,77] reported the degradation of atrazine herbicide in humic substances (HS) aqueous solutions and distilled water solutions on a laboratory scale upon γ-irradiation from a C 60o source, which can achieve 90% degradation rate of atrazine. And they later use γ-irradiation to degrade atrazine present in natural ground waters on a laboratory scale.

In 2015, Khan et al. [78,144] studied the kinetics, degradation pathways, influence of hydrated electron and radical scavengers in the degradation of aqueous atrazine by γ-irradiation, and the degradation rate can reach 69% under optimal conditions.

In addition, electron beams induced degradation of atrazine in aqueous solution was reported by Xu et al. [145] in 2015. Atrazine can be almost completely degraded (95%) and completely mineralized without any residue of cyanuric acid in aqueous solution.

## 5. Degradation Pathways, Atrazine Mineralization and Metabolites Toxicity

**Figure 3:**
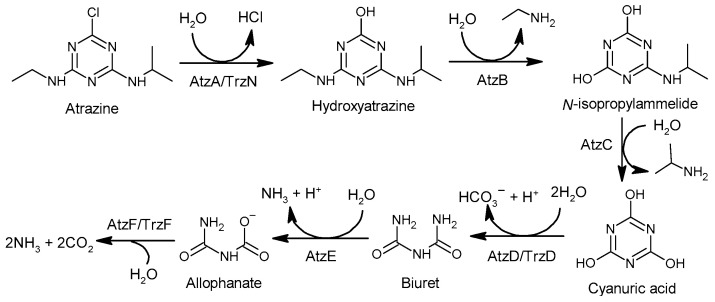
Degradation pathway of atrazine through biotic treatment process.

The degradation of atrazine is a complex process with different pathways through different biotic or abiotic water treatment processes. Regarding the biotic degradation processes, there are two stages [146] (Figure 3). In the first stage, hydrolytic dichlorination and *N*-dealkylation of atrazine generate cyanuric acid in the role of the enzymes that have broad substrate specificity [147]. For hydrolytic dichlorination of atrazine, enzyme atrazine chlorohydrolase (AtzA) [148] or hydrolase triazine (TrzN) [149] catalyzes hydrolytic dichlorination of atrazine, but they display substantial differences in their substrate ranges: AtzA is restricted to atrazine analogs with a chlorine substituent at carbon 2 and N-alkyl groups, ranging in size from methyl to t-butyl [150], and TrzN hydrolyzes a range of leaving groups (e.g., OCH_3_, –SCH_3_, –Cl, –F, –CN) from both triazines and pyrimidines [149]. For *N*-dealkylation of atrazine, hydroxyatrazine *N*-ethylaminohydrolase (AtzB) [151] catalyzes the hydrolytic conversion of hydroxyatrazine to *N*-isopropylammelide, and *N*-isopropylammelide isopropylaminohydrolas (AtzC) [152] catalyzes the hydrolysis of *N*-isopropylammelide to cyanuric acid. In the second stage, cyanuric acid is converted to ammonium and carbon dioxide by a set of enzymes AtzDEF [153,154] and TrzD [153,155].

The above discussion is based on the enzymatic steps catalyzed by the gene products. In actual operation, atrazine degradation may be achieved by a consortium of organisms harboring the appropriate combination of enzymes, for example, the enriched mixed culture as well as the isolated strain, designated as *Arthrobacter* sp. strain GZK-1, mineralized ^14^C-ring-labeled atrazine up to 88% to ^14^CO_2_ in a liquid culture within 14 d [156].

**Figure 4:**
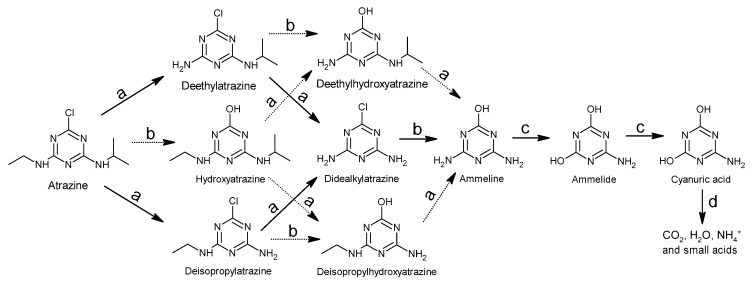
General involved degradation mechanisms of atrazine: (**a**) dealkylation of the amino groups; (**b**) dechlorination and hydroxylation of the s-triazine ring; (**c**) oxidation of the amino groups and deamination; (**d**) the opening of the s-triazine ring.

In addition, for abiotic water treatment processes, as shown in Section 2 and Section 3 of this article, many advanced oxidation processes (AOPs) have been involved in the degradation of atrazine in water. These AOPs can be used individually or in combination to improve efficiency such as US/UV [71,157], US/UV/O_3_ [114,158], electrochemistry (EC)/O_3_ [111], UV/H_2_O_2_ [159], UV/US/PS [160], UV/MW [161,162], UV/Fenton [83], etc. Generally, AOPs rely on the in situ formation of reactive species [78], such as hydroxyl radical (^•^OH) [163], sulfate radical (SO_4_^•−^) [164,165], singlet oxygen (^1^O_2_) [132], superoxide radical anions (O_2_^•−^) [37], hydrated electron (e_aq_^−^) [78] and hydrogen radical (H^•^) [78]. These reactive species have different redox potential and reaction selectivity. Therefore, the degradation pathways of atrazine vary from different AOPs. The general involved mechanisms were de-chlorination, hydroxylation of the s-triazine ring, de-alkylation of the amino groups, oxidation of the amino groups, de-amination and the opening of the s-triazine ring [71] (Figure 4). In most previous works [71,92,114,132,165], the final products of atrazine degradation tend to be cyanuric acid, ammelide and ammeline, because it is difficult to cleave the s-triazine ring [166]. At present, few studies [45,75,81,167,168] have reported the complete mineralization of atrazine, in which s-triazine ring-cleavage produced the less toxic compound biuret [167], and biuret hydrolyzed to allophanate, followed by the final generation of CO_2_, H_2_O, NH_4_^+^ and small acids. The complete mineralization of atrazine thus reduces the toxicity of the treated wastewater for subsequent release.

Toxicity studies on atrazine degradation are still incomplete, because some atrazine metabolites such as ammeline lack toxicological data. According to the book “Pesticide residues in food: 2007, toxicological evaluations”, published by the World Health Organization [169], atrazine, and its chloro-s-triazine metabolites are of moderate or low acute oral toxicity in male rats (LD_50_), 1870–3090, 1890, 2290 and 3690 mg/kg bw for ATZ, DEA, DIA and DDA, respectively; and the acute oral toxicity of hydroxyatrazine in male rats (LD_50_, >5050 mg/kg bw) is lower than that of atrazine or its chlorometabolites. However, toxicity comparisons based on these LD_50_ values are still inaccurate, as the results of toxicity tests vary based on different subjects (plants, animals, human cells, etc.) or different concerns (reproductive or developmental toxicity, liver toxicity, etc.). More toxicity tests data are shown above (Table 8). Combining these data, the following toxicity ranking can be roughly obtained: atrazine (ATZ) > deethylatrazine (DEA) > deisopropylatrazine (DIA) > ammeline (AM) > didealkylatrazine (DDA) > hydroxyatrazine (HA).

In addition, Banghai Liu et.al. [90] used the ECOSAR program to predict the acute and chronic toxicity of atrazine and its transformation intermediates, and it was found that although the vast majority of detected products possessed lower toxicity compared to atrazine, they remained classified as very toxic compounds to aquatic organisms.

The degradation mechanism, atrazine degradation rate, mineralization rate and main products are different for different treatment process. For better elaboration, the following discussion is based on treatment process type.

**Figure 5:**
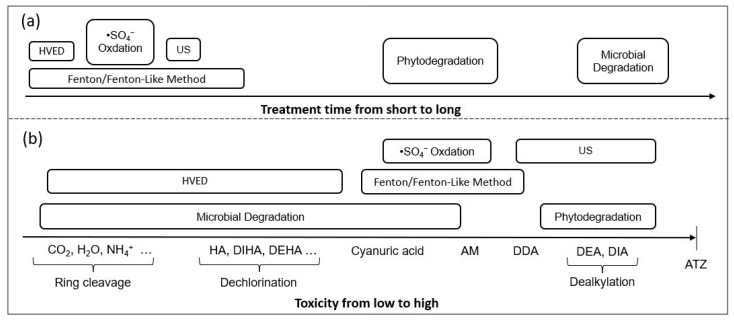
Comparison of different methods: (**a**) treatment time (**b**) product distribution.

As Table A1 shows, generally, different methods can achieve high degradation rates (>90%) of atrazine by filtering the optimal conditions, but the treatment time needed and atrazine degrading capacity vary. Figure 5 is a comparison of treatment time and atrazine product distribution of different methods based on the data listed in Table A1. We can see that the processing time required for biodegradation is significantly more than other methods, while HVED and Fenton/Fenton-like method take less time (Figure 5a). In addition, ring cleavage can be achieved by microbial degradation as well as HVED (Figure 5b). Compared with the Fenton method, HVED has the advantages of short processing time, high atrazine degrading capacity and low toxic product distribution.

## 6. Conclusions

As a widely used herbicide, atrazine is widely sprayed on many crops. Atrazine remaining in agri-food can cause physiological toxicity for a long time if it is ingested by humans. Additionally, because of chemical stability, atrazine in agri-food washing water flows into surface or groundwater and persists to be difficult to degrade. It is therefore of significant interest to develop clean and economical degradation processes for atrazine.

At present, biological processes are the common methods for the degradation of aqueous atrazine due to environmental protection, but biodegradation has its own limitations, such as slow degradation kinetics, and low remediation efficiency. Therefore, many studies have been focused on more highly efficient treatment technologies of aqueous atrazine, especially advanced oxidation processes (AOPs) that generate powerful nonspecific oxidant, hydroxyl radicals OH. Previous research reported the treatment of aqueous atrazine using ·OH generated by physicochemical methods and chemical methods. In these methods, a single technology processing or a co-processing of two or more technologies will be used, and often the latter can achieve a more ideal degradation rate. In addition to pursuing a high atrazine degradation rate, it is also significant to improve the degradation ability to achieve full mineralization. Therefore, more and more innovative technologies have been investigated, especially High Voltage Electrical Discharge (HVED). However, these new methods for the degradation of atrazine are still being explored, and further research is needed.

## Figures and Tables

**Table 1:** Generation methods of $SO_{4}^{-}\cdot$ for atrazine removal.

Generation Methods	Removal Effect
Carbon sheet fabricated from corn straw and potassium oxalate activated persulfate.	97.2% of atrazine was removed by the system within 20 min, when the concentration of persulfate was 2 mM [34].
Biochar supported nZVI composites (nZVI@BC) activated persulfate.	The atrazine removal rate was up to 93.8% [35].
Siderite/$CaSO_{3}$ system was used to provide $Fe^{2+}$ to activate sulfite.	>90% atrazine was removed within 6 min at 45 °C [36].
Pyrite activated persulfate.	100% of atrazine was degraded in 45 min and the TOC(total organic carbon) removal efficiency was 26% within 7 h [37].
Mechano chemically synthesized S-ZVI^bm^ composites activated persulfate.	The degradation of atrazine was up to 90%, which was pH-independent [38].
Nanoscale LaFe1-xCuxO3-δ perovskite activated peroxymonosulfate.	Atrazine (23 μM) was removed completely within 60 min in the presence of 0.5 g/L catalyst and 0.5 mM peroxymonosulfate [87].
Composite of nanoscale zero valent iron and graphene activated persulfate.	92.1% of atrazine was removed within 21 min using mass ratio of 5:1 nanoscale zero-valent iron (nZVI) to graphene (GR) [88].
Natural negatively-charged kaolinite with abundant hydroxyl groups activated peroxymonosulfate.	When the kaolinite dosage increased to 1.0 g/L, the degradation of atrazine exceeded 90% at 60 min [89].
Cobalt-impregnated biochar activated peroxymonosulfate.	99% of atrazine was degraded within 6 min [90].
Co-doped mesoporous $FePO_{4}$ activated peroxymonosulfate.	100% of atrazine was degraded for CoFeP-0.1 after 30 min at pH = 7 [91].
LaCoO_3_/Al_2_O_3_ activated peroxymonosulfate.	Under the optimal conditions, the removal rate and mineralization efficiency of ATZ reached 100% and 30.8%, respectively [92].
Copper sulfide activated persulfate.	The degradation of atrazine was up to 91.6% [93]
Hydroxylamine drinking water treatment residuals activated peroxymonosulfate.	The removal efficiency of atrazine was 95.5% in 30 min [94].
Fe_3_O_4_-sepiolite activated persulfate.	71.6% of atrazine and 20% of solution TOC were removed after 60 min [95].
CoMgAl layered double oxides activated peroxymonosulfate.	The degradation of atrazine was up to 98.7% [96].

**Table 3:** Electrocatalytic oxidation of aqueous atrazine.

Electrodes	Removal Effect
Co/Sm-modified Ti/PbO_2_ anode	The maximum degradation rate of 92.6% and the chemical oxygen demand (COD) removal rate of 84.5% are achieved in electrolysis time 3 h [44].
Fly ash-red mud particle electrode	90.1 % atrazine was degraded in 30 min [45].
Bifunctional nickel foam composite cathode co-modified with CoFe@NC and CNTs	The removal of atrazine reached 100% in 105 min under the given conditions, the removal efficiency of TOC after 420 min was 78.7 ± 2.6% [46].
Boron Doped Diamond (BDD) anode	Around 100% removal rate of atrazine was achieved in 4 h [47].
BDD anode	Permanganate was in situ electrochemical generated for the treatment of atrazine. Atrazine degradation increased significantly with permanganate production [48].
BDD anode	A high mineralization rate of 82% was obtained [18].
BDD, Carbon Felt, and Mixed Metal Oxides Anodes with Iridium and Ruthenium	BDD completely removes atrazine, and rest of anodes reached approximately 75% atrazine removal [109].

**Table 5:** Microbial degradation of aqueous atrazine.

Strain	Origin	Removal Effect
*Arthrobacter* sp. DNS10	Black soil [54]	The removal rate of 100 mg/L atrazine reached 95% and 86% in 0.05 mM Zn^2+^ and 1.0 mM Zn^2+^, respectively at 48 h [55].
*Bacillus badius* ABP6	Maize fields	Response-surface-methodology (RSM) was used to optimize environmental factors such as pH, temperature, agitation speed and atrazine-concentration on atrazine degradation by utilizing *Bacillus badius* ABP6 strain. In the optimum conditions (pH 7.05, temperature 30.4 °C, agitation speed 145.7 rpm, and atrazine-concentration 200.9 ppm), the degradation rate of atrazine reaches a maximum value of 90% [56].
*Bjerkanderaadusta*	Rotten wood surfaces	In the optimum conditions (pH 4, temperature 28 °C, biomass 2 g, and atrazine-concentration 50 ppm), the removal rate of atrazine was up to 92% in 5 days [57].
*Agrobacterium* sp. *WL-1*, *Arthrobacter* sp. *ZXY-2*	Jilin Pesticide Plant	After adding biochar ZXY-2 pellets, the removal rate of atrazine reached 61% within 1 h, higher than that treated by ZXY-2 pellets without biochar. The addition of biochar could enhance the connection between ZXY-2 and pellets-based carrier, and the favorable biodegradation pH of ZXY-2 changed to 6 and 10 [58].
*Chlorella* sp.	The Freshwater Algae Culture Collection at the Institute of Hydrobiology, China	Atrazine with initial concentration of 5 mg/L was photocatalytic degraded for 60 min with degradation ratio of 31%. After an 8 d exposure of the microalga *Chlorella* sp., 83% and 64% of the atrazine were removed from the degraded solutions containing 40 μg/L and 80 μg/L of atrazine, respectively [124].
*Myriophyllum spicatum*	Wuhan Botanical Garden	*Myriophyllum spicatum* absorbed more than 18-fold the amount of atrazine in sediments and degraded atrazine to hydroxyatrazine (HA), deelthylatrazine (DEA), didealkylatrazine (DDA), cyanuric acid (CYA) and biuret. The formation of biuret suggested for the first time, the ring opening of atrazine in an aquatic plant. The residual rate of atrazine was 6.5 ± 2.0% in *M. spicatum*-grown sediment on day 60 [125].

**Table 6:** Phytodegradation of aqueous atrazine.

Plant	Gene/Enzymes	Result
Pennisetum cladestinum	Soil dehydrogenase	Within 80 days, nearly 45% of atrazine was degraded [59].
Rice	Two novel methyltransferases LOC_Os04g09604, LOC_Os11g15040	Atrazine degradation and detoxification are regulated [62]
Alfalfa (Medicago sativa)	Genes encoding glycosyltransferases, glutathione S-transferases or ABC transporters	Atrazine in alfalfa can be detoxified through different pathways [63].

**Table 7:** Comparison of energy efficiency for the three pulsed electrical discharge processes.

Technology	Concentration of Atrazine (M)	Energy Efficiency (mol/J)
Pulsed electrical discharge in water [66]	2 × 10^−5^	3 × 10^−9^
Pulsed corona discharges above the water surface [64]	0.12 × 10^−3^	7.67 × 10^−10^
Pulsed arc electrohydraulic discharge in water [65]	2 × 10^−6^	1.56 × 10^−10^

**Table 8:** Chemical structures and toxicity tests data of atrazine and its metabolites.

Name	Atrazine (ATZ)	Deeth-Ylatrazine (DEA)	Deisoprop-Ylatrazine (DIA)	Ammeline (AM)	Cyanuric Acid	Dideal-Kylatrazine (DDA)	Hydroxy-Atrazine (HA)
Chemical structure	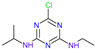	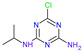	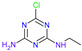	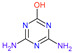	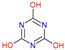	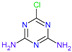	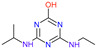
Acute oral toxicity in male rats (LD_50_) [169]	1870–3090 mg/kg	1890 mg/kg	2290 mg/kg			3690 mg/kg	>5050 mg/kg
Median lethal concentrations (LC_50_) for *Pseudokirchneriella subcapitata* in 96 h of exposure [170]	1600 μg/L	2000 μg/L	>3000 μg/L				
Concentration for 50% of maximal effect (EC_50_) on algal photosynthesis for *A. variabilis* [171]	0.1 ppm	0.7 ppm	4.7 ppm			100 ppm	>100 ppm
Acute oral toxicity in rats (LD_50_) [171]					>5000 mg/kg		
Adverse effects in sheep [172]				An average daily intake of ammeline 296 mg/kg body weight per day for 42 days for sheep caused half death.	No adverse effects at doses from 198 to 600 mg/kg body weight per day for 77 days.		

## Data Availability

Not applicable.

## Appendix A

**Table A1:** Comparison of different methods (degradation mechanism, atrazine degradation rate, atrazine mineralization rate and main products).

Method	Degradation Mechanism	Strain/Plant/Generated Reactive Species	Initial Atrazine Concentration and Some Notes	Treatment Time	Atrazine Degradation Rate	Atrazine Degrading Capacity	Products	References
Microbial Degradation	Microbes’ express atrazine-degrading enzymes that degrade atrazine.	*Chelatobacter heintzii* Cit1	The initial atrazine concentration is 0.5 mg per kg of soil. The bacteria described were isolated from 12 cultivated and grassland soils from different areas in France.	131 days	No residual atrazine detected	Ring cleavage	CO_2_, H_2_O …	[153]
		*Chelatobacter heintzii* Sal1-3						
		*Chelatobacter heintzii* LR3-3						
		*Chelatobacter heintzii* LRA						
		*Chelatobacter heintzii* SalB						
		*Chelatobacter heintzii* Lous2-3			56%	Dechlorination	Dechlorination products	
		*Chelatobacter heintzii* Sal2			No residual atrazine detected	Dechlorination	Dechlorination products	
		*Pseudomonas* sp. ADP				Ring cleavage	CO_2_, H_2_O …	
		*Arthrobacter cristallopoietes* Cit2				Cyanuric acid production	Cyanuric acid	
		*Nocardioides* sp. SP12						
Phytodegradation	Phytoextraction: atrazine in soil and groundwater can be taken up inside plant tissues; Phytotransformation: atrazine inside plant tissues can be transformed by plant enzymes; Rhizoremediation: pollutants in soil can be degraded by microbes in the root zone.	Tall fescue Ryegrass Barley Maize	49 days after planting, the soils were spiked with aqueous solutions of atrazine to achieve concentrations of 2, 5 and 10 mg of atrazine per kg of soil. The plants were harvested after 65 days, that is, 16 days after atrazine application.	16 days	88.6–96.7%	Dealkylation	DIA and DEA	[173]
					96.6–99.6%			
					96.4–99.4%			
					97.2–98.6%			
Fenton/Fenton-like Method	H_2_O_2_ reacts with Fe^2^^+^ to generate reactive radicals ^•^OH, which degrade atrazine.	^•^OH	The optimal mixture, 2.69 mM (1:1) FeSO_4_:H_2_O_2_, degraded [2,4,6-^14^C]-atrazine (140 µmol).	≤30 s	100%	Dealkylation	DDA	[174]
			The photo-Fenton process: 10 mg/L atrazine was degraded, using 1 g/L Heterogeneous Fenton catalyst Fe/TiO_2_, 1.6 mM H_2_O_2_ and pH = 3. The light intensity at 420 nm was 30 W/m^2^.	30 min	95%	Ring cleavage (TOC removal rate 18%)	DDA, Cyanuric acid …	[81]
			The initial concentration of atrazine was 23 µmol/L. The electro-Fenton process: the simultaneous reduction in ferric ions and oxygen at a simple electrode allowed the subsequent production of ^•^OH.	4 h	100%	Dealkylation	DDA	[29]
Sulfate Radical (SO_4_^•−^) Oxidation	With the activation of persulfate (PS), sulfate radical (SO_4_^•−^) can be generated by the cleavage of O–O bond of PS. Meanwhile, SO_4_^•−^ could react with water and OH^−^ to produce hydroxyl radicals (^•^OH).	SO_4_^•−^ and ^•^OH	The initial concentration of atrazine was 50 µmol/L. Copper sulfide (CuS)/persulfate (PS)	40 min	91.6%	Dealkylation and Dechlorination	AM	[93]
			The initial concentration of atrazine was 10 mmol/L. Magnetite Fe_3_O_4_-sepiolite/ persulfate (PS)	1 h	72.3%			[95]
			The initial concentration of atrazine was 20 mg/L. Pyrite (FeS_2_) /persulfate (PS)	45 min	100 %			[37]
High Voltage Electrical Discharges (HVED)	HVED can not only generate radical species, such as ^•^OH, HO_2_^•^, and H^•^, ions, and free electrons (e^−^), but also generate physical agents, such as UV, shock waves, and heat.	^•^OH	The initial concentration of atrazine was 11.9 mg/L. Dielectric barrier discharge (DBD)	18 min	93.7%	Ring cleavage (TOC removal rate 12.7%)	Dechlorination products, CO_2_, H_2_O …	[67]
Ultrasound (US)	The high energy generated by the collapse of the ultrasonic cavitation bubble leads to the generation of hydroxyl radicals (^•^OH) and hydrogen radicals (^•^H).	^•^OH	The initial concentration of atrazine was 0.1 mmol/L. Ultrasound frequency: 500 kHz	80 min	100%	Dealkylation	DEA, DIA, DDA	[70]
